# Supplementary material for: Patterns of Differentially Expressed circRNAs in Human Thymocytes
Source: Noncoding RNA. 2022 Mar 30;8(2):26. doi: 10.3390/ncrna8020026 (PMC9030292; doi:10.3390/ncrna8020026)
Supplement: Supplementary file 1 [file Supplementary Information Rebuttal.pdf › Supplementary Information Rebuttal.pdf]

non-coding RNA

MDPI

Supplementary Information

Patterns of differentially expressed circRNAs in human thymocytes

Pilar López-Nieva[1,2,*,c], Pablo Fernández-Navarro[3,4,*], María Ángeles Cobos-Fernández[1,2], Iria González-Vasconcellos[1,2], Raúl Sánchez Pérez[5], Ángel Aroca[5] José Fernández-Piqueras[1,2] and Javier Santos[1,2]

[1]Genome dynamics and function program, Genome decoding department, Severo Ochoa Molecular Biology Center (CBMSO), CSIC-Madrid Autonomous University, Madrid 28049, Spain.

[2] Institute of Health Research Jiménez Díaz Foundation, Madrid 28040, Spain.

[3] Cancer and Environmental Epidemiology Unit, National Center for Epidemiology, Institute of Health Carlos III, Madrid 28029, Spain.

[4] Consortium for Biomedical Research in Epidemiology and Public Health (CIBERESP), Madrid 28029, Spain.

[5] Department of Congenital Cardiac Surgery, Hospital Universitario La Paz, Madrid 28046, Spain.

Pilar López-Nieva, pilar.lopez@cbm.csic.es , https://orcid.org/0000-0001-6369-2444
Pablo Fernández-Navarro, pfernandezn@isciii.es, https://orcid.org/0000-0001-9427-2581
María Ángeles Cobos-Fernández, macobos@cbm.csic.es.
Iria González-Vasconcellos, iria.gonzalez@cbm.csic.es, https://orcid.org/0000-0002-4688-5508
Raúl Sánchez Pérez, raulcaravaca@hotmail.com
Ángel Aroca, aarocap@telefonica.net
José Fernández Piqueras, jfpiqueras@cbm.csic.es, https://orcid.org/0000-0003-4520-6785
Javier Santos, jsantos@cbm.csic.es, https://orcid.org/0000-0002-4168-6251

PLN and PFN contributed equally to this work

[c] Correspondence: pilar.lopez@cbm.csic.es; Tel.: +34 91 196 46 53

Supplementary Figure and Tables

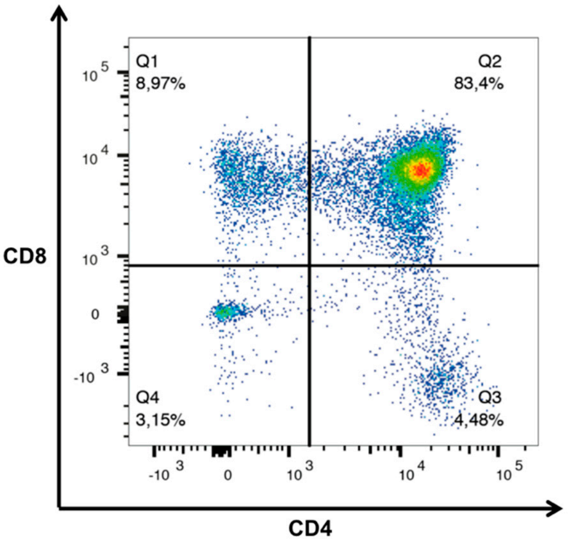

Figure S2. Representative flow cytometry analysis expression of CD4 and CD8 in human postnatal thymocytes. SP8: CD8+CD4− (Q1); DP: CD4+CD8+ (Q2); SP4: CD4+CD8− (Q3). DN: CD4-CD8− (Q4). Quantitative data above the plots represent mean absolute values ± SD of three independent experiments. Test were performed using GraphPad Prism version 9.3.1 for Mac OS X, (GraphPad Software, San Diego, California USA, www.graphpad.com)

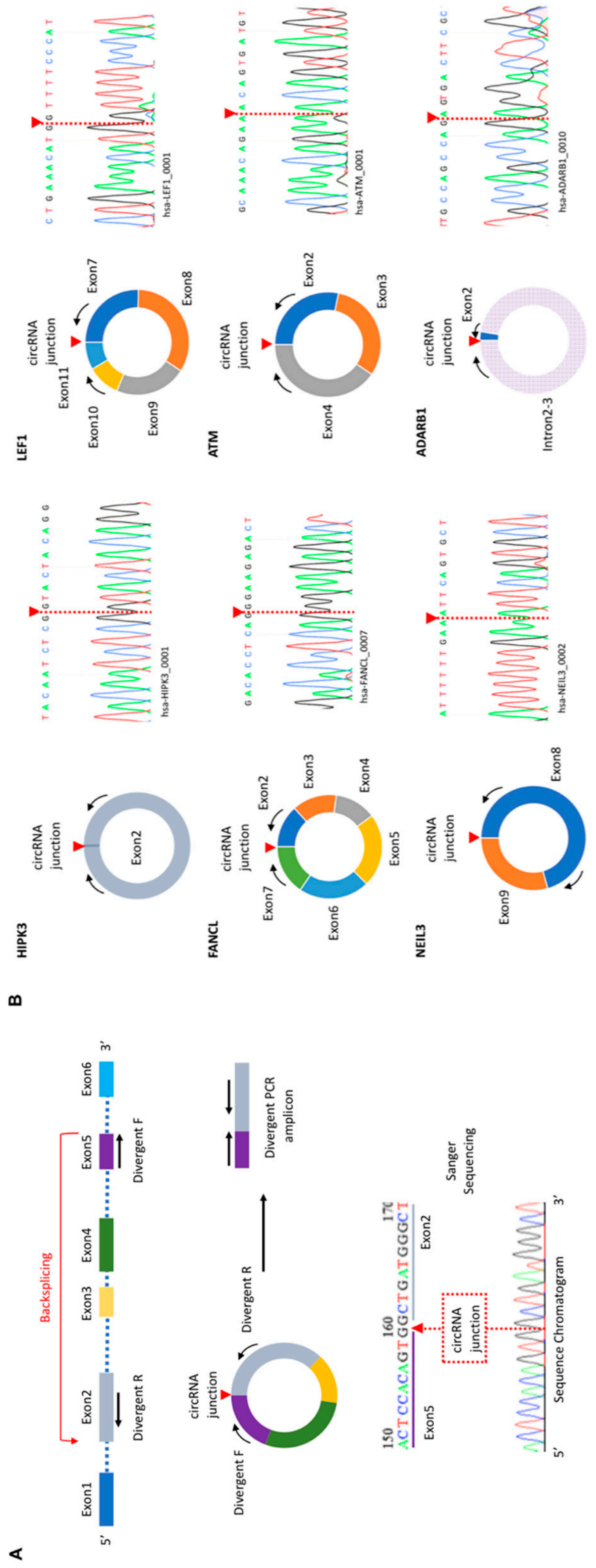
A
Backsplicing
5'
Exon1
Exon2
Exon3
Exon4
Exon5
Exon6
3'
Divergent R
Divergent F
circRNA junction
Divergent F
Divergent R
Divergent PCR amplicon
Sanger Sequencing
150
160
Exon5
Exon2
circRNA junction
Sequence Chromatogram
B
HIPK3
Exon2
circRNA junction
hsa-HIPK3_0001
FANCL
Exon2
Exon3
Exon4
Exon5
Exon6
Exon7
hsa-FANCL_0007
NEIL3
Exon8
Exon9
hsa-NEIL3_0002
LEF1
Exon7
Exon8
Exon9
Exon10
Exon11
hsa-LEF1_0001
ATM
Exon2
Exon3
Exon4
hsa-ATM_0001
ADARB1
Exon2
Intron2-3
hsa-ADARB1_0010

Figure S1.- Validation of circRNAs by qRT-PCR and Sanger sequencing. (A) Schematic illustration of circRNA biogenesis from backsplicing of pre-mRNA and schematic representation of the divergent primers used for detection and quantification of circRNAs. The red arrowhead represents the backsplice site. **(B)** Validation of circRNA expression using divergent primers. PCR products amplified with divergent primers resolved on Ethidium bromide-stained, 2% agarose gels. Sanger sequencing of purified PCR products showing the backsplice junction sequences of mentioned circRNAs. The red arrowhead represents the backsplice site.

Table S1. Patient characteristics table.

HTC (Human Thymus Code)	Age	Sex	Cytometric phenotyping (identification of cell populations in flow cytometry)			Cell Pool	Comments
			CD34+	DP (CD4+CD8+)	SP (CD4+ or CD8+)		
21017	1 week	Male	0,34%	79%	19,55%	DP/SP	Non chromosomal abnormalities, oncologic processes or genetic conditions with a propensity to develop them
241017	14 weeks	Male	0,23%	61,50%	35,50%	SP	Shone's complex. Non chromosomal abnormalities, oncologic processes or genetic conditions with a propensity to develop them
261017	20 weeks	Male	3,89%	85,10%	12,64%	DP/CD34/SP	Tetralogy of Fallot. Non chromosomal abnormalities, oncologic processes or genetic conditions with a propensity to develop them
81117	4 years y 2 moths	Female	3,42%	83,40%	13,45%	SP	Non chromosomal abnormalities, oncologic processes or genetic conditions with a propensity to develop them
131117	4 weeks	Male	3,32%	55,10%	34,50%	CD34	Non chromosomal abnormalities, oncologic processes or genetic conditions with a propensity to develop them
201117	2 weeks	Male	3,29%	74,70%	22,92%	CD34/SP	Non chromosomal abnormalities, oncologic processes or genetic conditions with a propensity to develop them
271117	2 weeks	Female	0,69%	79,40%	19,74%	DP/SP	Non chromosomal abnormalities, oncologic processes or genetic conditions with a propensity to develop them

Table S1. Patient characteristics table.

Table S2. Pairwise comparisons between circRNAs of the three thymocyte populations. 50 circRNAs are differentially expressed in at least one pairwise comparison (ST2 vs ST1, ST3 vs ST1 and ST3 vs ST2).

Table S3. Sequences of the DNA primers used to in this study.

Convergent (conv) primers

Gene Symbol		(5′–3′) sequences	Size (bp)
ADARB1	F	ACACCCTCATTCATCCAGCG	124
	R	GGTTTCTTGACTGGCGGAGA	
ATM	F	TGCGTGGCTAACGGAGAAAA	121
	R	ATCACTGTCACTGCACTCGG	
FANCL	F	TGGACACCTCAGAGCTCCTT	141
	R	TGCACTCCGTGGAGGTTTTT	
HIPK3	F	CAGTCTTCCTTCTCCGCTCC	166
	R	CTTCCTTCCCGGGGATTTGG	
IKZF1	F	GTGAAGTCCACACTGGCGTA	182
	R	GGGAGGTACGTTGTGCTGAA	
LEF1	F	CATGTCCAGGTTTTCCCATC	180
	R	TGAGGTCTTTTTGGCTCCTG	
NEIL3	F	TGTTTGGTCCTCCTCTGTTTCA	122
	R	GCCAACAATGGAAAGATGGCA	

Divergent (div) primers for circRNA detection and quantification

CircRNA ID	Gene Symbol		(5′–3′) sequences	Size (bp)
hsa-ADARB1_0010	ADARB1	F	TGAGCACACCCTCATTCATC	142
		R	AGTTGCCCCTTAAGCTCTCC	
hsa-ATM_0001	ATM	F	AGGCAGAAAAAGATGCAGGA	133
		R	ACGGCAGCAGATAAGCAGAT	
hsa-FANCL_0007	FANCL	F	TTTTCCTGTTCCATTTTGTGC	124
		R	TGTTCTCAGCTGCCAACTACA	
hsa-HIPK3_0001	HIPK3	F	GGGTCGGCCAGTCATGTATC	106
		R	ACTGCTTGGCTCTACTTTGAGT	
hsa-IKZF1_0001	IKZF1	F	GATGAGCCCATGCCGATCC	180
		R	GGGACATGTCTTGACCCTCA	
hsa-LEF1_0001	LEF1	F	CTTTATCCAGGCTGGTCTGC	229
		R	GTCAGTGTGGGGATGTTCCT	
hsa-NEIL3_0002	NEIL3	F	CCGAAAACAGCCCAATACTC	121
		R	CGGGTACTTCATTAAGTGGCTAA	

Table S3. Sequences of the DNA primers used to in this study.

Table S4. Comparison between fold change data between the different populations (ST1.ST2/ST1-ST3/ST2-ST3) and qPCR data

	Group	ANOVA (Tukey's Multiple Comparison Test Results) Difference	Lower	Upper	p-value Adjusted	log2FC
**hsa-HIPK3_0001**	**ST1-ST2**	-14,267	-17,099	-11,435	**0***	-1,59
	**ST1-ST3**	-13,267	-16,099	-10,435	**0***	-1,41
	**ST2-ST3**	0,1	-0,1832	0,3832	0,5573	0,15
**hsa-FANCL_0007**	**ST1-ST2**	-15,167	-19,247	-11,087	**0,0001***	-2,64
	**ST1-ST3**	-14,033	-18,113	-0,9953	**0,0001***	-1,5
	**ST2-ST3**	0,1133	-0,2947	0,5213	0,6871	0,73
**hsa-NEIL3_0002**	**ST1-ST2**	22,933	19,686	2,618	**0,0000015619***	1,41
	**ST1-ST3**	-0,33	-0,6547	-0,0053	**0,047***	-1,74
	**ST2-ST3**	-26,233	-2,948	-22,986	**0,0088381***	-3,18
**hsa-LEF1_0001**	**ST1-ST2**	20,167	18,742	21,591	**0,00022628***	3,54
	**ST1-ST3**	0,78	0,6375	0,9225	**0,000006564***	1,9
	**ST2-ST3**	-12,367	-13,791	-10,942	**0,0065563***	-1,37
**hsa-IKZF1_0001**	**ST1-ST2**	0,89	0,604	1,176	**0,0002***	1,61
	**ST1-ST3**	1,67	1,384	1,956	**0,0000044038***	2,47
	**ST2-ST3**	0,78	0,494	1,066	**0,0004**	0,83
**hsa-ATM_0001**	**ST1-ST2**	0,62	0,4495	0,7905	**0,0001***	2,24
	**ST1-ST3**	2,79	26,195	29,605	**0,000038172***	3,6
	**ST2-ST3**	2,17	19,995	23,405	**0,00063075***	0,98
**hsa-ADARB1_0010**	**ST1-ST2**	-0,1667	-0,4128	0,0795	0,175	1,51
	**ST1-ST3**	0,7	0,4538	0,9462	**0,0003***	3,48
	**ST2-ST3**	0,8667	0,6205	11,128	**0,0001***	1,39

Pairwise comparisons between circRNAs of the three thymocyte populations.

Table S4. Comparison between the fold change data between the different populations (ST1.ST2/ST1-ST3/ST2-ST3) and qPCR data.

Table S5. Differentially expressed mRNAs involved in T-cell differentiation.

Table S6.- Overexpressed as well as inhibited mRNAs detected during differential expression analysis for the three comparisons. The total mRNA detected was 16,032 (90.1%) in the whole set of samples. From the mRNA count data, differential expression analysis was performed using the Wald statistic and fold-change values were adjusted using the apeglm shrinkage estimator. In addition, an internal independent filter, parametric model fitting, normalization by ratio and including developmental stage (ST1/ST2/ST3) as a factor were applied. For the multiple comparisons problem, the p-value was adjusted using the BH procedure controlling the type I error rate, being $p < 0.05$ with a |log2FC| >= 1 and its standard error (SE), gene name and type of regulation (up/no/down) are shown. In addition, this table collects Ensembl biotype functional annotation, description of genomic elements, information contained in NCBI and GO terms of biological processes, molecular functions and cellular components associated with these described mRNAs and metabolic pathway information (KEGG) and PharmGKB. Information contained in this document: FeatureID, unique Ensembl identifier code of the genomic element analyzed; Filtering, identification of the genomic elements that have passed or not the independent filtering; Regulation, identification of the significant up/down regulated genomic elements; baseMean, normalized mean of counts for all samples; log2FC, logarithm in base 2 of the FC; SE.log2FC, standard error of the logarithm in base 2 of the FC; p.value, unadjusted significance level; p.adjusted, significance level adjusted by BH (FDR 5%); labid], count of replicates; Gene.Symbol, Gene symbol; Chr, chromosome where the genomic element is located; Band, cytoband; Biotype, classification of the genomic element detected in its Ensembl biotype; Gene.Synonyms, other aliases of the gene symbol; Description, NCBI gene information of the detected genomic element; KEGG.Pathways, identifier of KEGG metabolic pathways associated to a genomic element; GO.ID, identifier of ontological terms associated to a genomic element.

Table S7.- circRNA-miRNA-mRNA networks. Sheet "Results": Description of the circRNA-miRNA-gene networks constructed. This table shows the circRNA, miRNA and Genes included in the networks constructed and information about and information on the evaluation of the networks in relation to the possibility of including circRNA acting as miRNA sponge (see manuscript) and genes involved in T-cell differentiation in the thymus. Information contained in this table: circBaseID for circRNA; miRbase ID for miRNAs; Gene_symbol; type of network=(2)Network including a circRNA very possibly acting as miRNA sponge, (1)Network including a circRNA possibly acting as miRNA sponge, (0)Network not including a circRNA acting as miRNA sponge; GO_T_cell_diff (YES/NO)=gene included in the network involved in T-cell differentiation in the thymus attending to the description of GO-ONTOLOGY; ST2_ST1, ST3_ST1, ST3_ST2= Checking variables of the criteria for the network classification shown in the variable "type" for each comparison (DOWN*= log2 of fold change observed in both differential expression circRNA and mRNA analyzes were lower than or equal to -1 and FDR values were <=0.05, UP*= log2 of fold change observed in both differential expression circRNA and mRNA analyzes were higher than or equal to 1 and FDR values were <=0.05, DOWN= log2 of fold change observed in both differential expression circRNA and mRNA analyzes were lower than or equal to -1 and FDR values were >0.05, UP= log2 of fold change observed in both differential expression circRNA and mRNA analyzes were higher than or equal to 1 and FDR values were >0.05, NO= in any other option); log2FC, logarithm in base 2 of the FC in each comparison for circRNAs expression and for mRNAs expression. **Sheet "circRNA"**: this table collects the annotation of the circRNAs included in the constructed circRNA-miRNA-mRNA networks and the results of the Pairwise comparisons in Differential Expression analysis between the three thymocyte populations for each circRNA. **Sheet "mRNA":** this table collects for each gene included in the constructed circRNA-miRNA-mRNA networks, functional annotation, description of genomic elements, information contained in NCBI and GO terms of biological processes, molecular functions and cellular components associated with these described mRNAs and metabolic pathway information (KEGG) and PharmGKB and the results of the Pairwise comparisons in Differential Expression analysis between the three thymocyte populations for each mRNA.
